# Vascular covered stent and video-assisted thoracoscopic surgery for Aortoesophageal fistula caused by esophageal fishbone: a case report

**DOI:** 10.1186/s13019-024-02610-4

**Published:** 2024-03-09

**Authors:** Jianfeng Chen

**Affiliations:** Department of Anesthesiology, West China Hospital, Sichuan University, No 37 Guoxue Alley, Wuhou District, Chengdu City, Sichuan Province PR China

**Keywords:** Aortoesophageal fistula, Esophagus, Foreign body, Thoracic aortic injury, Endovascular treatment

## Abstract

**Background:**

Aortoesophageal fistula (AEF) is a rare condition characterized by communication between the aorta and esophagus. AEF caused by an esophageal foreign body is even rare, and there is currently no recommended standard treatment protocol. We report a case of delayed aortic rupture after the endoscopic removal of a fish bone, which was successfully treated with a combined approach of vascular stenting and thoracic surgery.

**Case presentation:**

A 33-year-old man presented to the hospital after experiencing chest discomfort for 3 days following the accidental ingestion of a fish bone. Under endoscopic guidance, the fish bone was successfully removed, and the patient was subsequently admitted for medical therapy. On the fourth postoperative day, the patient suddenly developed hematemesis, and chest computed tomography angiography revealed the presence of an AEF. This necessitated urgent intervention; hence, thoracic surgery was performed and a vascular-covered stent was placed. Following the surgical procedure, the patient received active medical treatment, recovered well, and was successfully discharged from the hospital.

**Conclusions:**

In patients with esophageal perforation caused by foreign bodies, hospitalization for observation, computed tomography angiography examination, early use of antibiotics, and careful assessment of aortic damage are advised. Thoracic endovascular aortic repair and esophageal rupture repair may have benefits for the treatment of AEF.

**Supplementary Information:**

The online version contains supplementary material available at 10.1186/s13019-024-02610-4.

## Background

It is very common for patients to seek medical attention from emergency departments for foreign esophageal bodies, such as fish bones, and their associated complications [1]. Most esophageal foreign bodies can pass through the digestive tract spontaneously, whereas certain objects can be successfully removed using techniques such as flexible or rigid endoscopy [2]. However, the occurrence of AEF due to esophageal foreign bodies is extremely rare [3], and its management poses significant challenges. Currently, there are no unified principles or guidelines for handling such cases, and the prognosis of patients with AEF is generally poor. Thoracic endovascular aortic repair (TEVAR) is considered an effective method for controlling bleeding in patients with AEF. However, patients who undergo TEVAR alone historically have a higher risk of postoperative rebleeding and mediastinitis and a poor long-term prognosis [4]. Here, we present our successful experience of managing a patient with delayed aortic rupture and AEF by combining TEVAR with a multidisciplinary treatment approach.

## Case presentation

A 33-year-old man presented to the emergency department of our hospital on September 23, 2021, complaining of poststernal pain that had persisted for 3 days after the ingestion of a fish bone. The patient presented with a fever (maximum temperature of 37.6 °C) and exhibited no dysphagia, hematemesis, hematochezia, melena, or other symptoms.

**Fig. 1:**
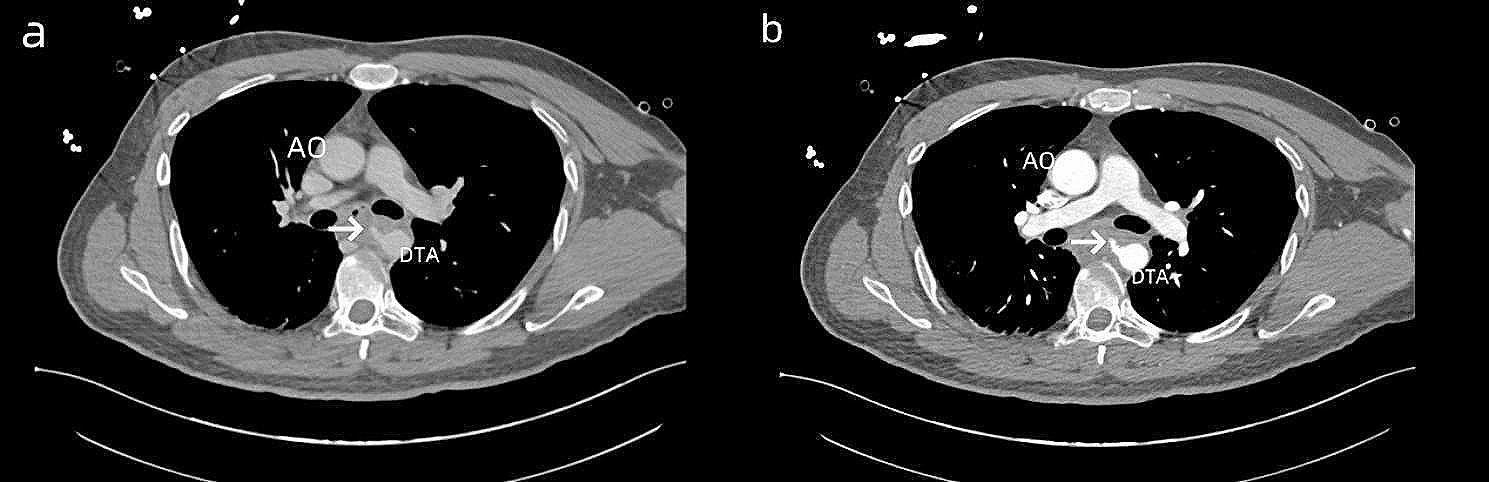
**(a, b)** Preoperative computed tomography images. CT showed the foreign body (white arrow) Ao: aorta, DTA:Descending Thoracic Aorta

A routine blood analysis showed a white blood cell (WBC) count of 13.57 × 10^9∕L. Emergency chest computed tomography angiography showed an oblique, strip-like, slightly high-density shadow in the middle segment of the esophagus (about the level of the thoracic 5–6 vertebrae), with a length of approximately 2.8 cm. Both ends of the foreign body had broken through the esophageal wall, with the front end reaching below the tracheal carina, about 0.5 cm from the right main pulmonary artery, and the back end about 0.1–0.2 cm from the thoracic aorta wall. There were multiple small air bubbles around the local esophagus, and no definite signs of contrast agent leakage were observed. This suggested foreign body perforation, with the back end close to the thoracic aorta (Fig. 1).

**Fig. 2:**
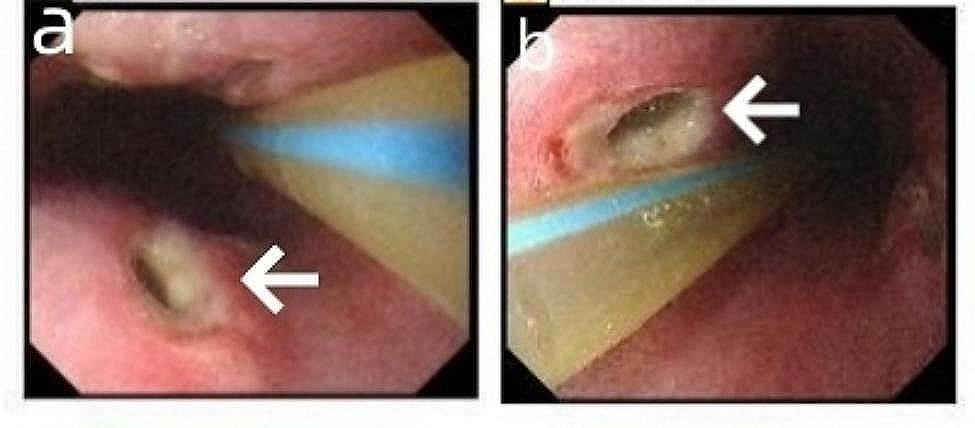
**(a, b)** Gastroscopic image. Esophageal perforation site (white arrow) after removal of fish bone

The patient underwent endoscopic extraction of the esophageal foreign body and insertion of a gastric tube under general anesthesia on September 24, 2021, at dawn. The linear foreign body was found deeply embedded in the esophageal wall 30 cm from the incisors, with deep ulcers formed on the right anterior and left posterior walls of the esophagus. The bottom of the ulcers was not detectable, and the foreign body was easily removed using forceps (Fig. 2).

Postoperatively, the patient was hospitalized for observation and symptomatic treatment. On September 29, 2021, at 01:20, the patient suddenly vomited approximately 1000 ml of bloody fluid with blood clots; his blood pressure dropped significantly, and tracheal intubation, fluid resuscitation, blood transfusion, and other measures to stabilize vital signs were immediately administered. An emergency chest CT contrast scan indicated esophageal perforation and partial rupture of the adjacent thoracic aorta. After relevant examinations confirmed that the patient had an esophageal-aortic fistula with massive hemorrhage, experts from multiple disciplines, including thoracic surgery, cardiac surgery, gastroenterology, and anesthesiology, collaborated closely to heal the patient. First, the cardiac surgeon performed endovascular repair of the aorta using a minimally invasive interventional technique. During the operation, a vascular stent-graft (HT2020-080-1500, Microport™) was placed in the lower segment of the descending aorta, and another vascular stent-graft (ETEW2424C82EE, Medtronic™) was placed in the upper segment of the descending aorta. After releasing the stent, no opacification of the aortic prominence or contrast extravasation was observed.

Subsequently, mediastinal abscess removal and esophageal rupture repair via the right thoracic under Video-Assisted Thoracoscopic Surgery(VATS) were performed. During surgery, a small amount of pale yellow pleural fluid was observed in the thorax, and a large number of blood clots were observed in the gap between the esophagus and aorta at the level of the carina, with a severe inflammatory response and dense adhesion of the surrounding soft tissues. A 1.5 cm break was observed on the posterior wall of the esophagus, with the esophageal mucosa exposed. The adventitia of the anterior wall of the aorta adjacent to the esophageal break was absent, and was suspected to be an aortic break. The esophageal rupture was sutured layer-by-layer with a 3 − 0 absorbable thread, and the azygos vein and connective tissue were sutured to the spaces around the esophagus and aorta to protect the aorta.

**Fig. 3:**
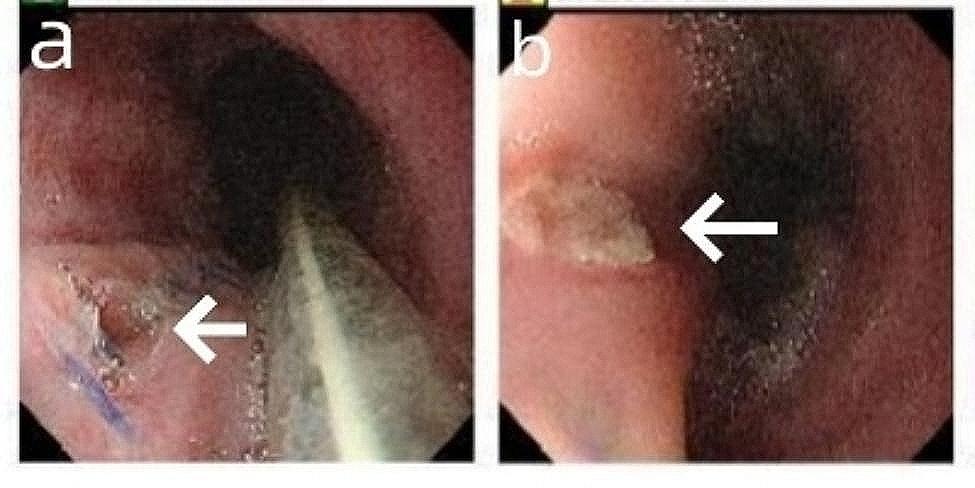
Postoperative gastroscopic image. Esophageal perforation site healed well (white arrow)

After surgery, the patient was administered anti-infection treatment (intravenous injection of piperacillin sodium tazobactam sodium for 3 weeks), jejunofeeding, and nasogastric decompression for 3 weeks, and was closely monitored for any recurrence of bleeding and fever. During this period, gastroscopy did not reveal any new bleeding at the site of the esophageal break (Fig. 3).

**Fig. 4:**
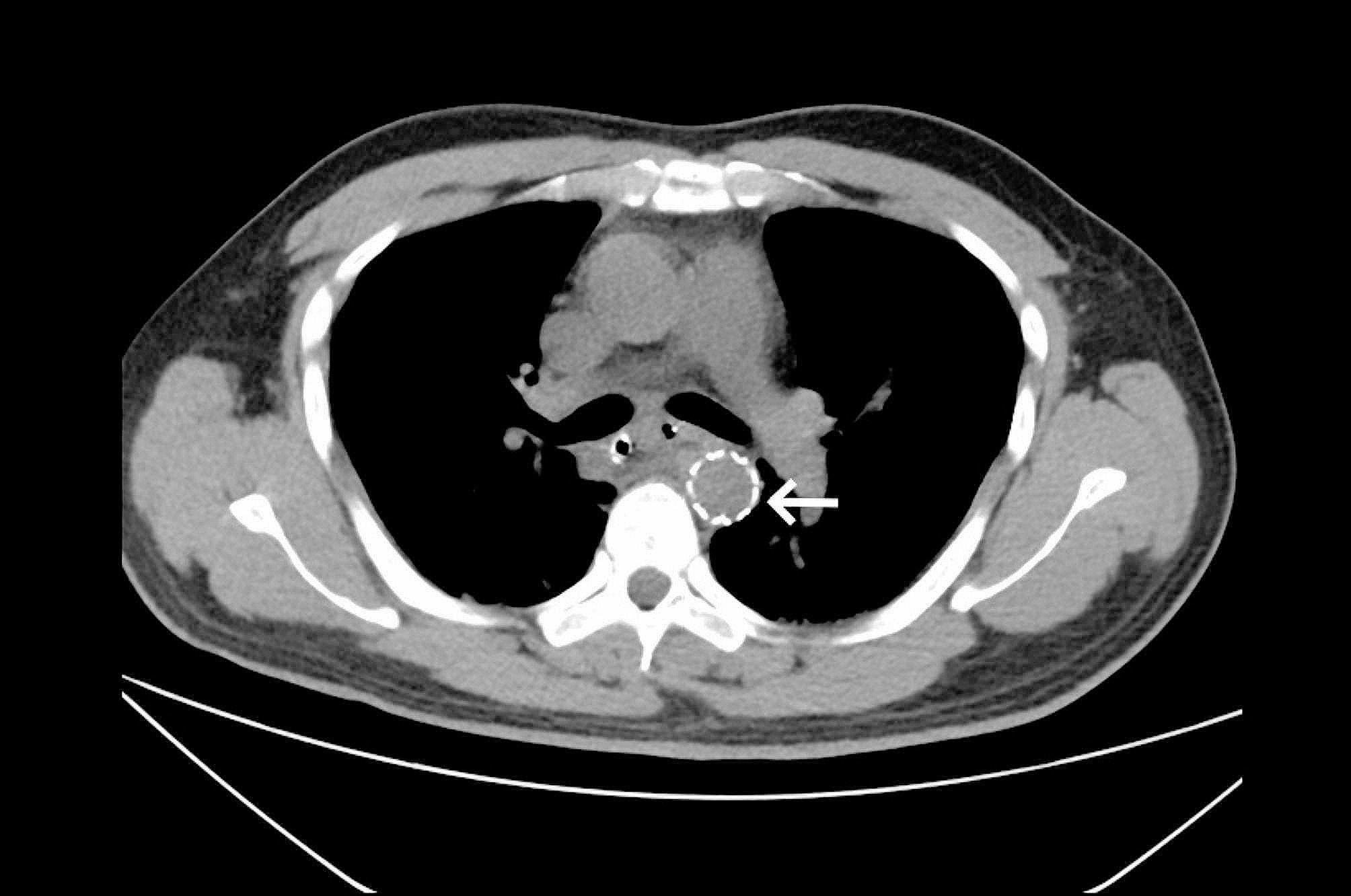
Computed tomography scan image. CT shows stent in descending aorta after surgery (white arrow)

Chest CT angiography (CTA) showed that the swelling at the esophageal break site had significantly improved (Fig. 4).

The patient began a liquid diet on postoperative day 12 and was discharged on day 29. The patient was confirmed symptom-free and alive at a one year follow-up after discharge.

## Discussion and conclusions

AEF refers to communication between the aorta and esophagus caused by esophageal or aortic lesions, which are common in aortic-related diseases [5]. AEF caused by an esophageal foreign body is very rare [6]. Due to differences in dietary structure and customs, esophageal foreign bodies in adults can vary from fish bones, pig bones, chicken bones or dentures [7]. The unique structure of fish bones makes them easy to pierce the surrounding structures of the esophagus and cause AEF. The typical symptoms of AEF are the Chiari triad, including chest pain, hematemesis, and subsequent massive upper gastrointestinal bleeding [8]. If a thrombus forms in the fistula, it may temporarily stop bleeding; however, persistent inflammation, fragile granulation tissue, and high pressure in the aorta interfere with the healing process [9]. At this time, performing esophagoscopy may cause unstable thrombus detachment and catastrophic bleeding [10]. When a patient has a clear history and typical manifestations, CTA is the preferred method of treatment [11]. After CTA showed the aorta was not damaged, the foreign body was removed under general anesthesia. Although severe esophageal rupture was found on endoscopy, there were no symptoms such as bleeding; therefore, the patient only received treatment of fasting, gastrointestinal decompression, and anti-infection treatment. Under such circumstances, the patient developed a delayed AEF during hospitalization, which is rare [12]. 

The treatment of AEF is challenging. Successful treatment was first documented in 1980, 160 years after the first reported case of AEF [13]. No standard treatment protocol for AEF has been established yet. Current treatment methods primarily include antibiotics and acid suppression, which have poor efficacy and high mortality rate [14]. Aortic replacement and TEVAR may be effective for AEF; however, the optimal treatment process remains controversial. Some studies have shown that patients who undergo TEVAR or aortic replacement alone may have a poor long-term prognosis due to complications such as infection, local inflammatory reactions, necrosis, and recurrent bleeding [15]. 

The prognosis of patients who have undergone esophageal reconstruction surgery is better than that of patients with esophageal stents [16]. However, there are still recent reports of good prognoses in patients with esophageal stents placed after TEVAR [17]. Today, whether TEVAR is considered a complete aortic replacement remains controversial, and it is unclear whether and when thoracic surgery is required [5]. Current studies are often based on retrospective analyses and case reports, which may be limitations. Due to the rarity of the disease, there are no multicenter, large-sample studies comparing the prognosis of patients following different treatment methods, which could be improved upon in the future.

AEF caused by an esophageal foreign body is essentially an infection in which the sterile mediastinum and aorta are contaminated by substances from the gastrointestinal tract [18]. The surrounding inflammation induces weakening and fragility of the aortic wall, which in this case may have caused the delayed AEF. Several days after eating fish bones, the patient presented with fever and elevated white blood cell count, at which point the inflammation had advanced [19], but CTA did not show significant damage or rupture of the aorta. Although the formation of ulcers were observed after the fish bone was removed under endoscopy, neither endoscopic treatment nor thoracoscopic exploration was performed. Once foreign bodies in the esophagus cause local abscesses and mediastinitis, conservative treatment alone (antibiotics, placement of gastric tubes, etc.) may not prevent the infection from invading the aortic wall, and may result in delayed aortic rupture. The only effective method to prevent delayed AEF may be performing thoracoscopic exploration to remove the abscess, repair or excise the perforated esophagus, and strengthen the esophagus and aorta with tissue fascia immediately after esophageal ulcer formation was found [20]. After the onset of delayed AEF, active thoracoscopic esophageal surgery and clearance of the thoracic abscess may have been necessary.

Although the patient survived the AEF, there were some limitations to this case. Due to the rapid AEF process, we did not have enough time to develop a well-prepared plan. It may be dangerous to perform TEVAR, considering the possibility of infection invasion, and that aortic replacement is not performed at a later stage. Studies have shown that infected fistulas of the aorta may lead to infection of the coated stent and cause serious complications. A variety of broad-spectrum antibiotics should be used in the early stages of the disease to improve survival rates [18]. Therefore, the progression of infection after early emergency hemostasis should be closely evaluated, and aortic replacement surgery should be actively performed when necessary to prevent stent infection and other serious consequences.

In conclusion, patients with suspected AEF resulting from an esophageal foreign body should be hospitalized for observation, undergo CTA examination, and be given early antibiotics and prompt assessment of the involvement of the mediastinum and aorta. Once AEF occurs, emergency TEVAR combined with thoracoscopic surgery can rapidly control its progression.

### Electronic supplementary material

Below is the link to the electronic supplementary material.

Supplementary Material 1

Supplementary Material 2

Supplementary Material 3

## Abbreviations

AEF: Aortoesophageal fistula
VATS: Video-assisted thoracic surgery
CT: Computed tomography
CTA: CT angiography
TEVAR: Thoracic endovascular aortic repair
WBC: White blood cell
