# Supplementary figures and images for: Vascular covered stent and video-assisted thoracoscopic surgery for Aortoesophageal fistula caused by esophageal fishbone: a case report
Source: J Cardiothorac Surg. 2024 Mar 9;19:112. doi: 10.1186/s13019-024-02610-4 (PMC10924337; doi:10.1186/s13019-024-02610-4)

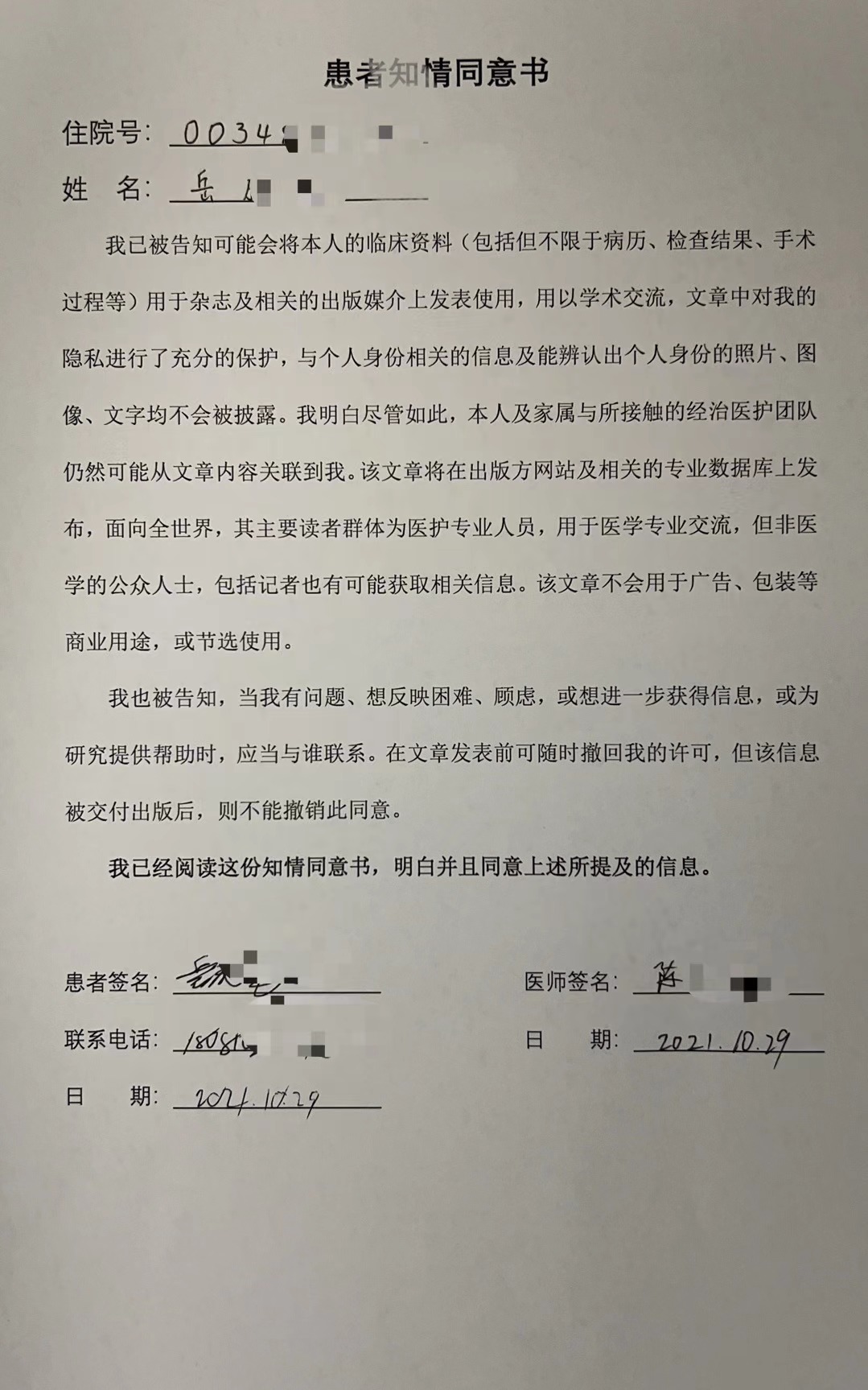

Supplement: Supplementary file 1 — Supplementary Material 1 [file 13019_2024_2610_MOESM1_ESM.jpg]
